# A Ten-Year Retrospective Survey of Antimicrobial Susceptibility Patterns among Salmonella enterica subsp. *enterica* Serovar Typhi Isolates in Ontario, Canada

**DOI:** 10.1128/spectrum.04828-22

**Published:** 2023-01-09

**Authors:** Shawn T. Clark, Kirby Cronin, Antoine J. Corbeil, Samir N. Patel

**Affiliations:** a Department of Laboratory Medicine and Pathobiology, University of Toronto, Toronto, Ontario, Canada; b Public Health Ontario, Public Health Ontario Laboratory, Toronto, Ontario, Canada; Johns Hopkins Hospital

**Keywords:** *Salmonella* Typhi, antimicrobial resistance, antimicrobial susceptibility testing, surveillance

## Abstract

The epidemiology and treatment of typhoid fever are complicated by the emergence and spread of Salmonella enterica subsp. *enterica* serovar Typhi lineages with resistance to many antimicrobial agents critical for therapy. Current information on the susceptibility patterns of S. Typhi isolates identified in regions where typhoid fever is not endemic is important as these are often acquired after traveling to countries of endemicity where resistant strains circulate. Here, we report a 10-year retrospective survey of S. Typhi antimicrobial susceptibility patterns among 858 unique patient isolates that underwent reference laboratory testing in Ontario, Canada, between 2010 and 2019. Antimicrobial susceptibility patterns remained stable for ampicillin (average, 78.7% susceptible), azithromycin (average, 99.4% susceptible) ertapenem (average, 100.0% susceptible), meropenem (average, 100.0% susceptible), and trimethoprim-sulfamethoxazole (average, 78.2% susceptible) during the study period; however, nonsusceptibility to ciprofloxacin and ceftriaxone increased. While ceftriaxone-resistant isolates comprised 1.6% of the total isolates overall, they represented 10.1% of the total isolates tested in 2019, indicating a significant increase over time. Our findings suggest that when selecting empirical therapy, health care providers should strongly consider current trends in antimicrobial susceptibility and investigate the patient’s exposure risk to gauge whether a suspected typhoid infection may be caused by a potentially resistant S. Typhi strain.

**IMPORTANCE** This work provides an updated summary of the antimicrobial susceptibility patterns among Salmonella Typhi strains isolated from patients in Ontario, Canada.

## INTRODUCTION

The incidence of antimicrobial-resistant bacterial infections continues to increase, with increasingly extensive drug resistance leading to fewer therapeutic options being available (1). Typhoid (or enteric) fever is a systemic febrile illness caused by the fecal-oral transmission of Salmonella enterica subsp. *enterica* serovar Typhi. In 2017, over 10 million individuals were estimated to have contracted typhoid fever worldwide, with over 100,000 cases being fatal (2). Selection pressure from antibiotic use in areas where typhoid fever is endemic has resulted in the global emergence of S. Typhi lineages with various degrees of resistance to first- and second-line typhoid antimicrobial agents, namely, ampicillin (AMP), chloramphenicol, trimethoprim-sulfamethoxazole (SXT), ciprofloxacin (CIP), third-generation cephalosporins, and azithromycin (AZM) (3–7). Of particular concern are extensively drug-resistant (XDR) strains identified in regions of South Asia and the Middle East that are resistant to at least five antimicrobial groups (8), usually including all of the agents mentioned above except azithromycin (3, 4, 9, 10). These strains can carry resistance-associated genes on plasmids and have the potential to disseminate and further propagate in susceptible bacterial populations (11).

In regions such as Canada where typhoid is not endemic, the majority of S. Typhi infections occur in travelers (10, 12, 13). Since 2019, the Public Health Agency of Canada has recommended a series of health precautions for those planning to travel to areas experiencing XDR S. Typhi outbreaks, including pretravel vaccination and personal hygiene methods for infection prevention during travel (14). Given its large and multiculturally diverse population, the majority of reported Canadian typhoid fever cases are in Ontario (see Results, below). As the burden of resistant S. Typhi strains increases in areas of endemicity, the extent to which this trend impacts the empirical management of infections diagnosed in areas where typhoid fever is not endemic remains to be defined (15). A previous survey of S. Typhi isolates in Ontario between 2002 and 2007 reported that a high proportion of isolates were resistant to nalidixic acid (NAL), a surrogate for fluoroquinolone resistance, and advised against the use of ciprofloxacin for empirical therapy (16). Between 2018 and 2019, 10 XDR S. Typhi isolates related to the Pakistan XDR S. Typhi outbreak strain were identified from pediatric and adult patients in Ontario, prompting the provincial recommendation of using carbapenems or macrolides for the empirical management of suspected typhoid infections if an individual had traveled to Pakistan (13, 17). A similar recommendation was published by the U.S. Centers for Disease Control and Prevention (CDC) in 2021 (18). Since 2007, an up-to-date and comprehensive assessment of S. Typhi resistance in the Ontario population has not been performed.

The purposes of this study were to (i) retrospectively survey antimicrobial susceptibility patterns among S. Typhi isolates tested at Ontario’s provincial reference laboratory over a 10-year period, (ii) determine whether provincial- and local-level susceptibility patterns are reflective of the global trends reported, and (iii) identify whether increased resistance to certain antimicrobial drugs may hamper their use empirically.

## RESULTS AND DISCUSSION

A total of 1,587 confirmed cases of typhoid fever were reported in Canada between 2010 and 2019 via the Canadian Notifiable Diseases Surveillance System (CNDSS) (average, 159 cases reported annually [range, 121 to 194]) (see Table S1 in the supplemental material). Cases in the province of Ontario accounted for approximately 55.0% of the total reported cases nationwide (Table S1). The Public Health Ontario (PHO) Laboratory received 1,003 S. Typhi isolates from 858 unique patients over the 10-year study period, representing >98% of confirmed cases in Ontario during this period. An average of 100 ± 27 isolates (range, 67 to 155) were tested annually at the PHO Laboratory from blood (77.0%), stool (20.8%), or other specimen sources (2.2%) (Table 1; Fig. S1).

**TABLE 1:** Demographics of patients with S. Typhi infections in Ontario, Canada, between 2010 and 2019

Demographic information	No. (%) of patients
Specimen source	
Blood	775 (77.0)
Stool	209 (20.8)
Urine	15 (1.5)
Other	7 (0.7)
Patient age distribution (yrs)	
0–5	121 (14.1)
6–17	202 (23.5)
18–65	514 (60.0)
>65	14 (1.6)
Not specified	7 (0.8)
Patient biological sex	
Male	424 (49.4)
Female	408 (47.5)
Not specified	26 (3.0)
Region of travel	
Africa	1 (1.7)
South Asia	54 (93.1)
North America	1 (1.7)
South America	1 (1.7)
≥1 region noted	1 (1.7)

After patient duplicates were removed, 858 S. Typhi isolates representing unique typhoid fever episodes were included in our retrospective analysis. There was no difference in the proportions of S. Typhi isolates tested by biological sex (Table 1). The median patient age was 23 years (range, 0 to 89 years) (Table 1) and did not differ by biological sex (*P* = 0.25 by Welch’s *t* test). Only 6.8% (*n* = 58) of the 858 unique patient isolates had a known travel history recorded on the test requisition submitted to the laboratory. Of those, most listed travel to South Asia (93.1%; *n* = 54), namely, India (74.1%; *n* = 43), Pakistan (10.3%; *n* = 6), and Bangladesh (8.6%; *n* = 5) (Table 1). With respect to the geographic distribution of cases within Ontario, 51.2% of individuals with S. Typhi infection resided in the highly populated Central Ontario Region as defined by Ontario Health (including Halton, Peel, and Simcoe-Muskoka) (Table S2).

**FIG 1:**
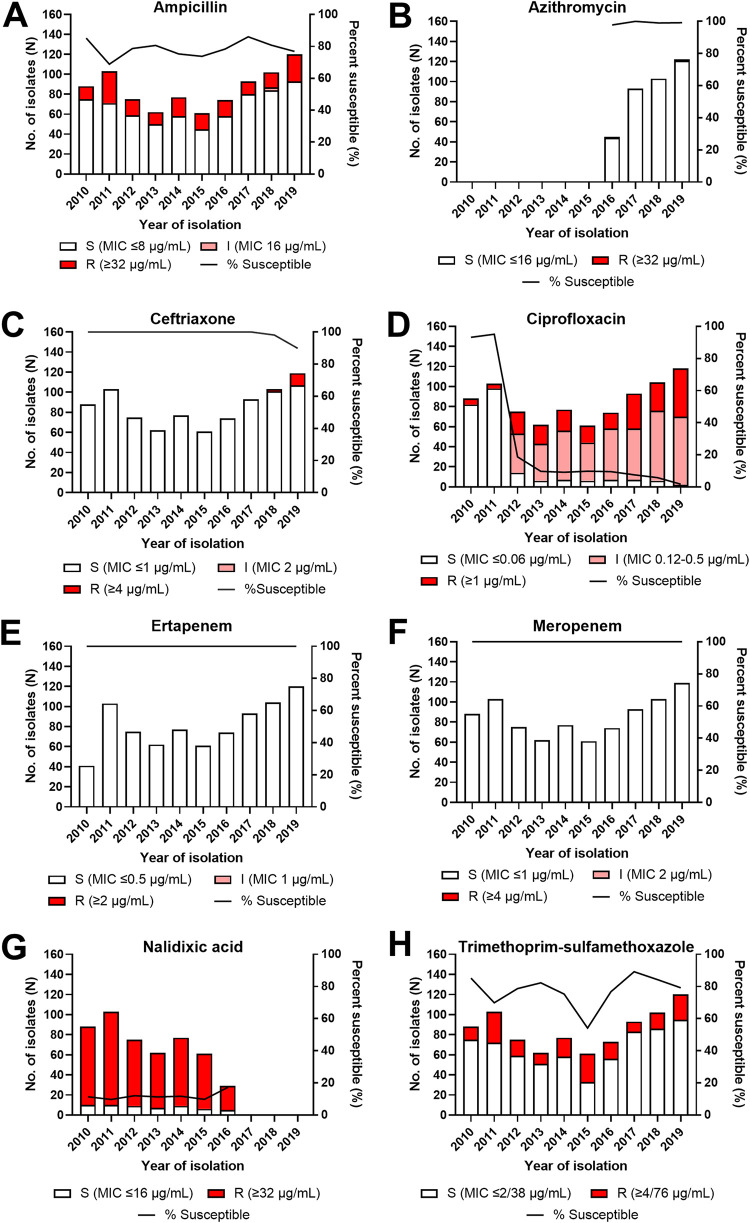
Antimicrobial susceptibility patterns of S. Typhi isolates in Ontario, Canada, between 2010 and 2019. Isolates (*n* = 855) were tested for susceptibility to ampicillin (A) azithromycin (B), ceftriaxone (C), ciprofloxacin (D), ertapenem (E), meropenem (F), nalidixic acid (G), and trimethoprim-sulfamethoxazole (H) by agar dilution and classified as susceptible (S), intermediate (I), or resistant (R) as appropriate. The trendline indicates the percentage of isolates considered susceptible based on 2022 CLSI interpretive criteria (26).

Moderate levels of resistance were noted among all S. Typhi isolates for the antimicrobial agents AMP (20.7%), CIP (25.4%), and SXT (21.8%) based on 2022 interpretive criteria from the Clinical and Laboratory Standards Institute (CLSI) (Table 2 and Fig. 1). Of the eight antimicrobials examined, significant increases in the numbers of nonsusceptible isolates (isolates with susceptibility results classified as intermediate or resistant) were identified for both ceftriaxone (CRO) (χ^2^ = 25.7; *P* < 0.0001) and CIP (χ^2^ = 352.0; *P* < 0.0001) over time. The increased likelihood of recovering isolates with reduced CIP susceptibility in the present day is not surprising (Fig. 1) and is in keeping with previous trends identified in Ontario by Morris and colleagues (16). This may reflect the global expansion of the S. Typhi H58 lineages that carry genotypic signatures of fluoroquinolone resistance (e.g., mutations in *gyrA*) following decades of empirical CIP use in the 1990s and 2000s (5, 7). Only two isolates with resistance to another oral agent, AZM, were identified in this study from two unique patients, one in 2016 and one in 2019. Both of these isolates were resistant to AZM and susceptible to CRO, ertapenem (ETP), and meropenem (MEM); however, the 2019 isolate was also resistant to AMP and CIP (data not shown). The development of resistance to AZM is of importance as it is often the only active oral agent remaining for XDR infections. Isolates with macrolide resistance have emerged independently in Bangladesh (19), India (6), Nepal (20), and Pakistan (3) in recent years.

**TABLE 2:** Antimicrobial susceptibility profiles determined for S. Typhi isolates tested in Ontario, Canada, between 2010 and 2019

Antimicrobial	No. of unique isolates tested^*a*^	MIC range tested (μg/mL)	% of isolates with susceptibility profile^*b*^			MIC_50_ (μg/mL)	MIC_90_ (μg/mL)
			S	I	R		
Ampicillin	855	≤8 to ≥32	78.7	0.35	20.7	≤8	≥32
Azithromycin	362	≤16 to ≥32	99.4	NA	0.6	≤16	≤16
Ceftriaxone	855	≤1 to ≥64	98.4		1.6	≤1	≤1
Ciprofloxacin	855	≤0.06 to ≥4	27.5	47.1	25.4	0.5	≥4
Ertapenem	810	≤0.25 to ≥0.5	100.0			≤0.5	≤0.5
Meropenem	855	≤0.12 to ≥1	100.0			≤0.12	≤1
Nalidixic acid	495	≤16 to ≥32	11.3	NA	88.7	≥32	≥32
Trimethoprim-sulfamethoxazole	854	≤0.5/9.5 to ≥8/152	78.2	NA	21.8	2/38	4/76

a Nalidixic acid testing was performed by the laboratory only until 2017.

b Determined based on criteria defined in CLSI document M100 (26). NA indicates that there was no breakpoint defined for a given antimicrobial agent. S, susceptible; I, intermediate; R, resistant.

Prior to 2018, CRO-resistant S. Typhi was undetected in the United States and Canada. The Canadian Antimicrobial Resistance Surveillance System first identified CRO resistance in isolates from 2018, where 2.9% of Canadian isolates were CRO resistant (21). A similar trend was observed by the U.S. CDC National Antimicrobial Resistance Monitoring System, where annual increases in CRO resistance were noted, from 2.7% in 2018 to 20% in 2020 (22). The current study reports a similar trend in Ontario, with CRO-resistant isolates first being detected in 2018, with a 6-fold increase in prevalence in 2019 to represent 10.1% of the total number of S. Typhi isolates that year (Fig. 1). The CRO resistance phenotypes among S. Typhi isolates that have been reported to date can be either sporadic or associated with XDR strains (17, 23), which reflects the increasing reliance on broad-spectrum agents such as carbapenems to empirically treat severe typhoid fever cases. There was no significant change in overall susceptibility for the remaining antimicrobials during the study period, with ETP and MEM being the most stable phenotypes observed.

Given that resistant S. Typhi isolates have been readily detected in some regions of South Asia, we also examined whether any of the 54 isolates from patients with a known history of travel to this region (based on laboratory requisitions only) had unique susceptibility profiles (Table S3). Among this group, we noted that isolates from Pakistan were often resistant to AMP, NAL, and SXT and had reduced susceptibility to CIP (Table S3). Only a single blood isolate from a patient who had traveled to Pakistan in 2018 displayed resistance to AMP, CIP, CRO, and SXT. This is in keeping with recent epidemiological trends seen in Pakistan, where multiple resistant lineages (including XDR strains) are reported to circulate (4, 9). Of interest, isolates from India collected after 2016 remained mostly susceptible to SXT. It is important to note that these descriptive exposure-linked data represented only a small fraction of our larger population size, which had no travel data available, and therefore may not be representative. A lack of travel exposure could also be significantly associated with an increased risk of resistance, as noted by the U.S. CDC in its 2021 health advisory in which 9 XDR S. Typhi cases had no reported travel or other epidemiological links (18). A more in-depth epidemiological analysis using reliable travel exposure history would assist in further delineating expected susceptibility patterns in patients exposed to different geographic locations.

Our study has several limitations. First, while our data set is large and represents almost all S. Typhi cases reported within the province of Ontario over a prolonged period, only a small number of isolates had accompanying clinical and exposure information provided, making robust inferences of anticipated susceptibility profiles per individual patient scenario not possible. This highlights one of the challenges with laboratory-based surveillance studies and the importance of submitters performing their due diligence and providing more complete epidemiological data (where available) for any isolates submitted for reference testing. Of note, not all infected individuals may have sought care, and although the relative burden of asymptomatic S. Typhi carriage in the population is likely limited, such cases may not have been captured in our data set. Although outside the scope of the current study, additional whole-genome sequencing and comparative genomic analyses of these isolates would have been beneficial in predicting genotypic resistance mechanisms and isolate relatedness and further characterizing the predominant lineages identified in Ontario.

In summary, this study provides an updated survey of antimicrobial susceptibilities among S. Typhi strains isolated from patients in Ontario, Canada. While antimicrobial susceptibility patterns remained fairly stable over the 10-year period, the increase in CRO-resistant S. Typhi since 2018 suggests that a careful balance between the use of increasingly resistant first-line options (e.g., CRO) and the use of alternative agents (e.g., AZM, ETP, and MEM) currently reserved for those with a high risk of XDR infection is needed, alongside prompt, antimicrobial susceptibility testing (AST)-tailored therapy once results are available.

## MATERIALS AND METHODS

### Retrospective data collection and susceptibility testing.

The Public Health Ontario (PHO) Laboratory provides reference microbiology services to public health units, hospitals, and community laboratories across the Canadian province of Ontario. Although not mandated, all S. Typhi isolates in Ontario (including primary specimens for laboratories performing culture-independent diagnostic testing only) are requested to be routinely sent to the PHO for confirmation, AST, and molecular typing surveillance (24). We collected and analyzed retrospective test data for all S. Typhi isolates submitted to the PHO Laboratory between 1 January 2010 and 31 December 2019. For each isolate, these data included patient demographic information (age, gender, year of specimen collection, specimen source, travel history, and local public health unit) and AST results for eight antimicrobials from seven classes: ampicillin (AMP), azithromycin (AZM), ceftriaxone (CRO), ciprofloxacin (CIP), ertapenem (ETP), meropenem (MEM), nalidixic acid (NAL) (not tested routinely and tested only until 2017), and trimethoprim-sulfamethoxazole (SXT). Only the first S. Typhi isolate per patient per study year was included, with any duplicates being excluded from the analysis. In cases where isolates from both a sterile (e.g., blood) and a nonsterile (e.g., stool) source were submitted from the same patient within the same time period, only the isolate from the sterile source was retained, and the isolate from the nonsterile source was excluded. To determine the total number of reported typhoid fever cases in Canada and Ontario between 2010 and 2019, we used publicly available national data from the Canadian Notifiable Diseases Surveillance System (CNDSS) (https://diseases.canada.ca/notifiable/charts-list) and publicly available provincial data from the Infectious Disease Trends in Ontario (IDTO) tool (https://www.publichealthontario.ca/en/Data-and-Analysis/Infectious-Disease/Reportable-Disease-Trends-Annually).

AST of all isolates against the eight antimicrobials mentioned above was performed by agar dilution according to Clinical and Laboratory Standards Institute (CLSI) methods (25). The MIC, the lowest concentration of an antimicrobial that inhibits bacterial growth, was interpreted for each antimicrobial using current breakpoints described in CLSI document M100 (26). The MIC_50_ and MIC_90_, the MIC values representing inhibition of 50% and 90% of isolates, respectively, for each antimicrobial were calculated as previously described (27).

### Statistical analysis.

Patient demographic information was analyzed using descriptive statistics where applicable. Comparisons between patients by age and biological sex were made using Welch’s *t* test, with a *P* value of ≤0.05 being considered significant. The total number of isolates classified as susceptible, intermediately resistant, or resistant to the eight antimicrobials was determined annually. Patterns of changes in susceptibility to a given antimicrobial were examined over time using the chi-square test for trends, with a *P* value of ≤0.05 being considered significant. All analyses were performed in GraphPad Prism 9 v9.3.1. (GraphPad Software LLC, La Jolla, CA, USA).
