# Supplementary material for: A Ten-Year Retrospective Survey of Antimicrobial Susceptibility Patterns among Salmonella enterica subsp. enterica Serovar Typhi Isolates in Ontario, Canada
Source: Microbiol Spectr. 2023 Jan 9;11(1):e04828-22. doi: 10.1128/spectrum.04828-22 (PMC9927447; doi:10.1128/spectrum.04828-22)
Supplement: Supplemental file 1 — Supplemental material. Download spectrum.04828-22-s0001.pdf, PDF file, 0.2 MB [file spectrum.04828-22-s0001.pdf]

Supplementary information

for

A Ten-Year Retrospective Survey of Antimicrobial Susceptibility Patterns Among *Salmonella enterica* subspecies *enterica* serovar Typhi Isolates in Ontario, Canada

Shawn T Clark, Kirby Cronin, Antoine J. Corbeil, Samir N. Patel

This document includes

Figure S1
Table S1
Table S2
Table S3

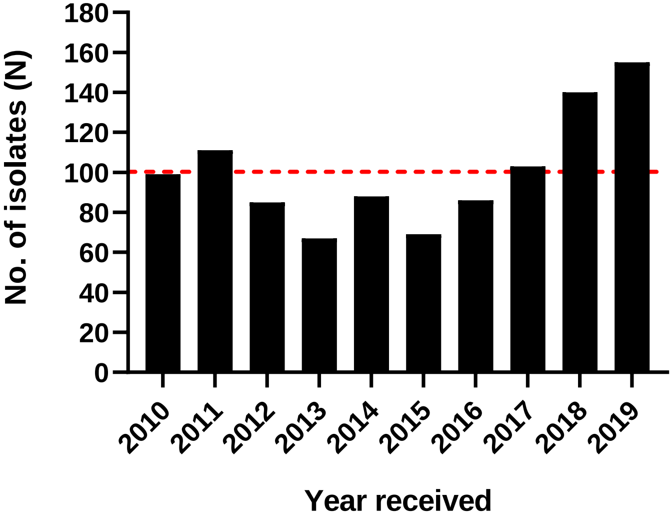

Figure S1. Number of *S*. Typhi isolates submitted annually for reference testing between 2010 and 2019 in Ontario, Canada (N=1,003). The hatched red line indicates the overall average number of isolates received per year (N=100.3).

Table S1. Cases of *S*. Typhi reported in Canada and Ontario between 2010 and 2019

Year	Number of reported cases (N)		Percentage of total reported Canadian cases identified in Ontario, Canada (%)
	Ontario	Canada	
2010	88	175	50.3
2011	103	186	55.4
2012	82	143	57.3
2013	60	128	46.9
2014	73	133	54.9
2015	64	121	52.9
2016	69	127	54.3
2017	104	190	54.7
2018	106	190	55.8
2019	122	194	62.9

Table S2. Regional reporting of *S.* Typhi isolates in Ontario, Canada (2010-2019)

Region[a]	Percentage of isolates (%(N))
Central	51.2 (439)
East	6.4 (55)
North	0.7 (6)
Toronto	31.1 (267)
West	10.6 (91)

[a] Denotes the geographic region to which the local public health units involved are located

The following local public health units were included:

Central - includes Halton, Peel, Simcoe-Muskoka

East - includes Durham, Hastings-Prince Edward County, Kingston, Ottawa, Perth, Peterborough

North - includes North Bay-Perry Sound, Northwestern, Sudbury, Thunder Bay

Toronto - includes the city of Toronto

West includes Brant, Chatham-Kent, Grey-Bruce, Hamilton Haldimand, Middlesex-London, Niagara, Oxford, Waterloo, Wellington-Dufferin, Windsor-Esse[illegible]

Table S3. Analysis of isolates from patients with known travel history to South Asia

Region of travel	Year	Source	AMP	AZT[a]	CTX	CIP	ERT	MEM	NAL[a,b]	TMP-SMX
Bangladesh	2012	Blood	R	-		I			R	R
	2013	Blood		-		I			R	
	2013	Blood	R	-		R			R	
	2015	Stool		-		I			R	
	2019	Blood				I			-	
India	2010	Blood		-					R	
	2010	Blood		-					R	
	2010	Blood		-					R	
	2010	Blood		-					R	
	2010	Stool		-					R	
	2010	Blood		-		R			R	
	2010	Blood		-		R			R	
	2011	Blood		-					R	
	2011	Blood		-					R	
	2011	Blood	R	-					R	R
	2011	Blood	R	-					R	R
	2011	Stool	R	-					R	R
	2012	Blood		-		R			R	
	2012	Blood		-		R			R	
	2012	Blood		-		I			R	
	2012	Blood		-		R			R	
	2013	Blood		-		I			R	
	2014	Blood		-		I			R	
	2014	Blood		-		I			R	
	2014	Blood		-		I			R	
	2014	Blood	R	-		I			R	R
	2014	Stool	R	-		I			R	R
	2015	Blood		-		R			R	R
	2015	Blood		-		R			R	R
	2015	Blood		-		I			R	R
	2016	Blood				R				
	2016	Blood				R				
	2016	Blood				I				
	2016	Stool				I				
	2016	Blood		-		I			R	
	2016	Blood	R			I				R
	2017	Blood				R			-	
	2017	Blood				R			-	
	2017	Blood				R			-	
	2017	Blood				I			-	
	2018	Blood				R			-	
	2018	Blood				I			-	
	2018	Blood				I			-	
	2018	Blood				I			-	
	2019	Blood				R			-	
	2019	Blood				I			-	
	2019	Blood				I			-	
	2019	Blood				I			-	
Pakistan	2011	Blood	R	-					R	R
	2012	Blood		-					R	
	2013	Blood	R	-		I			R	R
	2015	Blood	R	-		I			R	R
	2017	Blood	R			I			-	R
	2018	Blood	R		R	R			-	R

[a] An - indicates the antimicrobial was not tested on the isolate

[b] Only tested until 2017

(blank)	Susceptible
I	Intermediate
R	Resistant
